# Current Perspectives on “Off-The-Shelf” Allogeneic NK and CAR-NK Cell Therapies

**DOI:** 10.3389/fimmu.2021.732135

**Published:** 2021-12-01

**Authors:** Erica L. Heipertz, Evan R. Zynda, Tor Espen Stav-Noraas, Andrew D. Hungler, Shayne E. Boucher, Navjot Kaur, Mohan C. Vemuri

**Affiliations:** ^1^ Cell & Gene Therapy, Thermo Fisher Scientific, Frederick, MD, United States; ^2^ BioProduction, Thermo Fisher Scientific, Grand Island, NY, United States; ^3^ BioProduction, Thermo Fisher Scientific, Oslo, Norway

**Keywords:** natural killer cells, CAR-NK cells, immunotherapy, NK cell expansion, lentiviral delivery, AAV delivery, killer immune receptors, GMP manufacturing

## Abstract

Natural killer cells (NK cells) are the first line of the innate immune defense system, primarily located in peripheral circulation and lymphoid tissues. They kill virally infected and malignant cells through a balancing play of inhibitory and stimulatory receptors. In pre-clinical investigational studies, NK cells show promising anti-tumor effects and are used in adoptive transfer of activated and expanded cells, *ex-vivo*. NK cells express co-stimulatory molecules that are attractive targets for the immunotherapy of cancers. Recent clinical trials are investigating the use of CAR-NK for different cancers to determine the efficiency. Herein, we review NK cell therapy approaches (NK cell preparation from tissue sources, ways of expansion *ex-vivo* for “off-the-shelf” allogeneic cell-doses for therapies, and how different vector delivery systems are used to engineer NK cells with CARs) for cancer immunotherapy.

## Natural Killer Cell Biology

Human natural killer (NK) cells are innate cytotoxic lymphoid cells derived from CD34+ precursors originating from hematopoietic stem cells (1, 2) and play an essential role in tumor surveillance. Unlike T cells, NK cells can kill malignant cells in an antigen-independent manner and have shown promise in a number of clinical trials involving both solid and hematological cancers (3). NK cells do not require HLA matching. Their ability to act in an antigen-independent manner makes them a viable option for an “off the shelf” therapy that can be manufactured on a large scale and easily distributed to cancer patients.

NK cells are subdivided into two populations based on their relative expression of CD56 (neural cell adhesion molecule; NCAM) and CD16: immature CD56^bright^ CD16^neg^ NK cells, and mature CD56^dim^ CD16^pos^ NK cells (4). The CD56^bright^ population accounts for 10% of NK cells circulating in the blood and are located primarily in lymph nodes. Immature CD56^bright^ NK cells have an immunoregulatory function and produce cytokines, such as interferon-gamma (IFN-γ), TNFα, TNF-β, IL-10, and GM-CSF (5). In contrast, the mature CD56^dim^ CD16^pos^ population accounts for up to 90% of the circulating NK cells (6). The key function of mature CD56^dim^ CD16^pos^ NK cells is natural and Ab-mediated cell cytotoxicity. Mature CD56^dim^ NK cells express high amounts of killer cell immunoglobulin receptors (KIRs) (7).

The mechanism for the transition from CD56^bright^ to CD56^dim^ is still widely unknown, but the change in surface markers is a major indicator for transitioning to maturity (2, 7). CD16, CD27, CD56, CD57, and perforin are all markers for NK cell maturation (7, 8). CD27 is a marker of immature NK cells, associated with the TNFα receptor group and found on three times as many immature CD56^bright^ as mature CD56^dim^ (7). Inversely CD57 and perforin are markers for terminal maturity and are highly expressed on mature NK cells (7, 9).

Located throughout the body, NK cells represent 5-20% of all lymphocytes in the blood and organs with high concentrations in the bone marrow, spleen, liver, lungs, skin, kidneys, uterus, and secondary lymphoid tissue (8, 10). The tissue-specific location has been shown to have a significant impact on NK cell functionality and cytokine production. Mature NK cells in the lung are shown to produce higher amounts of granzyme B, a serine protease associated with cytotoxicity, than those NK cells found in the lymph nodes or gut (8).

NK cells secrete a number of pro-inflammatory cytokines, such as TNF and IFN-γ that stimulate an adaptive immune response and prevent tumor angiogenesis (5). The production levels of IFN-γ and TNFα from NK cells can be stimulated through various cytokines such as IL-12, IL-15, and IL-18 (8).

**Figure 1:**
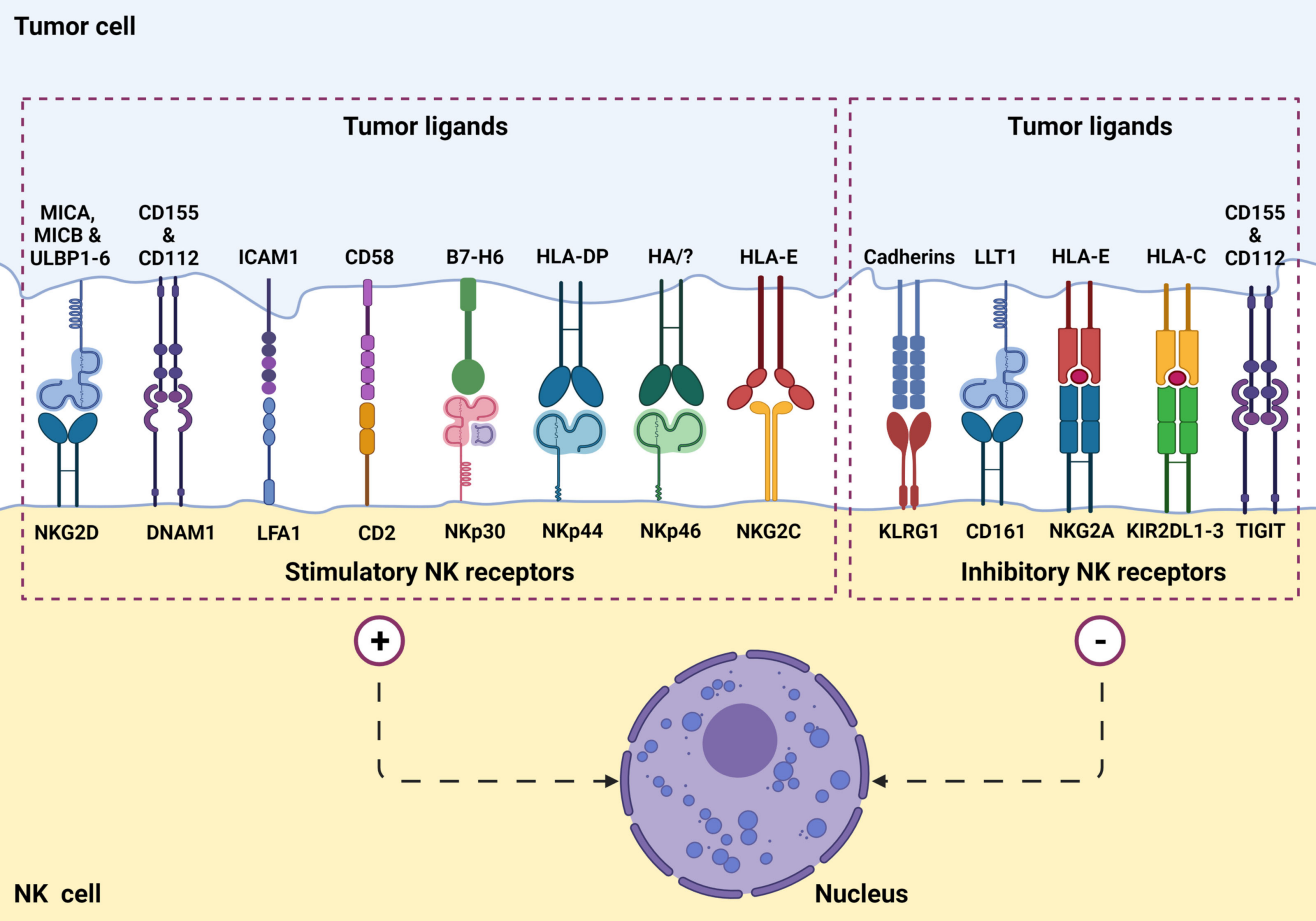
NK cell surface receptors and ligands on tumor cells are involved in tumor recognition. NK cells express a set of stimulatory (or activation) receptors as well as inhibitory receptors to recognize healthy cells and aberrant cells such as virus-infected or a potential tumorigenic cell through MHC-1 receptor appearance.

**Figure 2:**
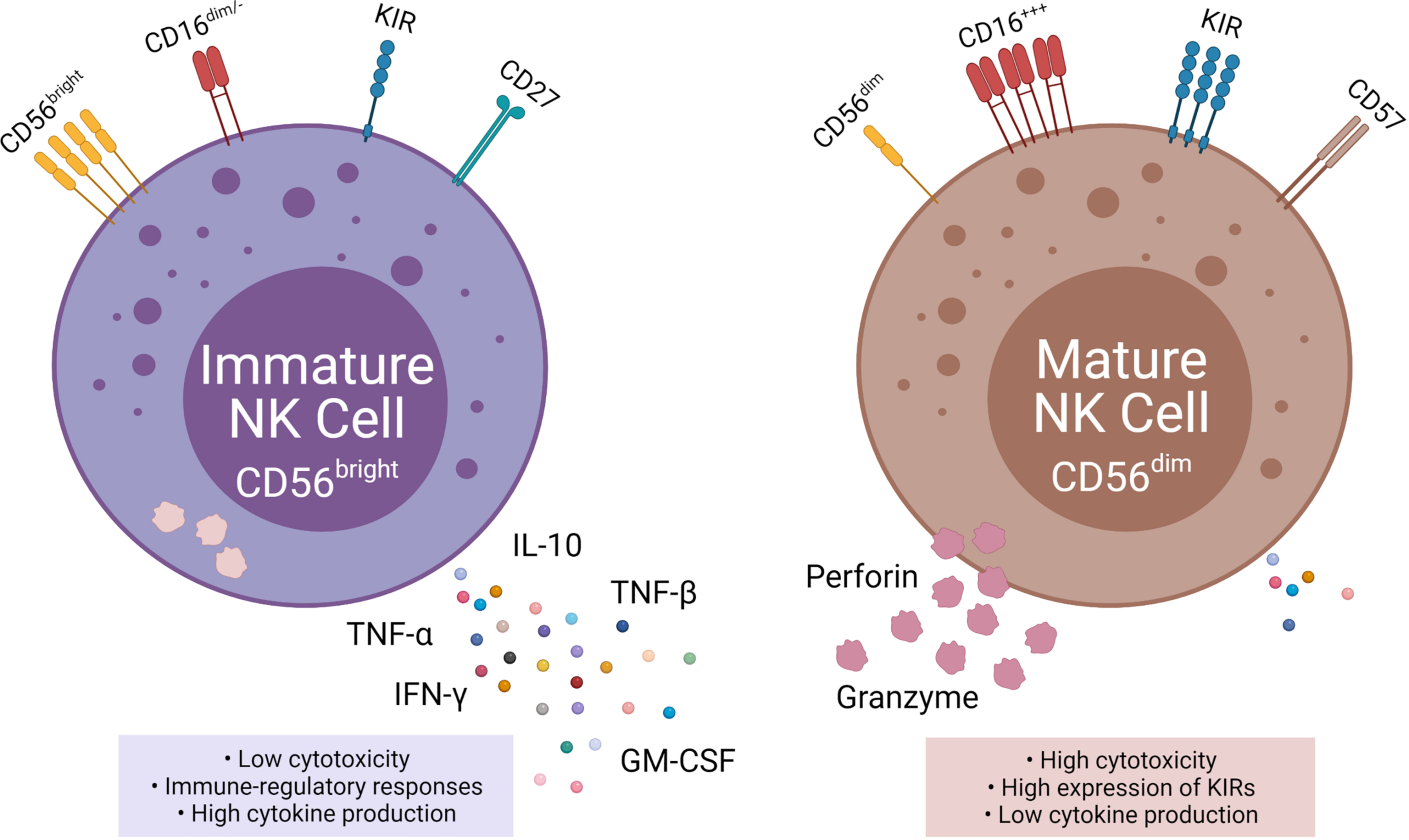
Phenotypic and functional properties of immature (left) and mature (right) NK cells. Immature NK cells express CD56^bright^, absent, or CD16^dim^, low KIR, and CD27 and are also known as NK^regulatory^ that exhibit low cytotoxicity, but high cytokine production. Mature NK cells, in contrast, express CD56^dim^, high CD16, high KIRs, and CD57 and are also knows as NK^cytotoxic^ that exhibit high cytotoxicity and low cytokine production.

NK cells function by killing virally infected, stressed, and cancerous cells in an antigen-independent manner (1, 2, 8). Additionally, NK cells work to activate other immune cells using co-stimulatory signals (2). NK cell’s cytolytic function is based on an array of activation and inhibitory signals (**Figure 1**) as well as self-major histocompatibility complexes (MHC) class I molecules (1, 2). NK cells recognize target cell MHC class I molecules which bind to the NK cell KIRs allowing the NK to identify “self.” This self-identification inhibits the cytotoxic activity against normal cells (1, 2, 7). In addition to preventing cytotoxic function, this binding also prevents inflammation and helps with the “licensing” of the immature NK cells (7). Tumor cells often downregulate MHC class I expression to avoid lysis by cytotoxic T cells. Additionally, DNA damage and cellular stress upregulates tumor ligands’ expression on malignant cells, which are recognized by NK cell-activating receptors (**Figure 2**). NK cells will trigger cell-mediated lysis (1) if a cell down-regulates its MHC class I molecules and upregulates activation ligands.

Once a cell is designated as infected, stressed, or cancerous, NK cells work to kill it through a direct release of cytolytic granules containing perforin and granzyme B. The contents of cytolytic granules are released from the cell *via* degranulation (**Figure 2**). The granules from the NK cell form a synapse with the target cell, releasing the cytolytic contents. Perforin and granzyme B are key components of cytolytic granules and trigger apoptosis through caspase-dependent and independent mechanisms. Perforin aids in the entry of the granzyme B into the target cell, which ultimately leads to target cell death (11).

In addition to direct lysis of malignant or virally infected cells, CD56^dim^ CD16^pos^ NK cells mediate antibody-dependent cellular cytotoxicity (ADCC). ADCC is triggered when NK cells recognize an antibody opsonized target cell. The binding of CD16 with the Fc portion of IgG antibodies trigger the release of perforin and granzyme B which lyse the target cell (11). ADCC is provoked by several therapeutic monoclonal antibodies (mAbs) and may enhance the homing and efficacy of NK cell therapy (12).

## NK Cell-Based Strategies in Clinical Trials Targeting Different Indications

Autologous and allogeneic NK cell therapies have shown great potential in preclinical studies and clinical trials. Different strategies are considered in clinical trials using NK cells for cancer therapies, including utilizing an agonist to NK cell activation receptors (mABs; transtuzumab, rituximab, etc., + IL-2 and anti PD1) or by blocking NK cells inhibitory receptor signals with mABs to KIR (NKG2A-CD94 or with CTLA-4 and PD-1 checkpoint inhibitor) (13). Recent findings demonstrate the potential of allogeneic NK cells for hematological malignancies and solid tumors (14). Unlike T cells, NK cells do not induce graft-*versus*-host-disease (GVHD) and their alloreactivity is enhanced under KIR mismatch with HLA ligands on cancer cells (15). Several clinical trials have highlighted the safety of the allogeneic transfer of NK cells (16). Allogeneic NK cells were used to target different cancers including hematological malignancies, lymphoma, leukemia, and solid tumors such as melanoma, neuroblastoma, gastric cancers, ovarian and breast tumors (**Table 1**).

**Table 1:** Completed allogeneic NK cell clinical trials.

	NCT Number	Title	NK Cell source	Status	Conditions	Interventions	Clinical trial phase	Population	Sponsor/ Collaborators	Dates	Locations / Outcome
1	NCT03358849	Phase 1 Clinical Trial to Evaluate the Safety of Allogeneic NK Cell ("SMT-NK") Cell Therapy in Advanced Biliary Tract Cancer	Not available	Completed: No results posted	• Advanced Biliary Tract Cancer	• Biological: Natural killer cell	Study Type: Interventional Phase: Phase 1	Enrollment: 9 Age: 18 Years to 75 Years (Adult, Older Adult) Sex: All	• Yonsei University	Study Start: October 17, 2017 rimary Completion: September 27, 2018 Last Update Posted: January 16, 2019	• Division of Gastroenterology, Department of Internal Medicine, Yonsei University College of Medicine, Seoul, Korea, Republic of
2	NCT02008929	to Evaluate the Efficacy and Safety of MG4101(Ex Vivo Expanded Allogeneic NK Cell)	Allogeneic expanded NK Cells	Completed: No results posted	• Hepatocellular Carcinoma	• Biological: MG4101	Study Type: Interventional Phase: Phase 2	Enrollment: 5 Age: 20 Years to 69 Years (Adult, Older Adult) Sex: All	• Samsung Medical Center	Study Start: August 2014 Last Update Posted: December 3, 2015	• Samsung Medical Center, Seoul, Korea, Republic of
3	NCT01212341	Allogeneic Natural Killer (NK) Cell Therapy in Patients With Lymphoma or Solid Tumor	Allogeneic NK Cells	Completed - No results posted	• Malignant Lymphomas • Solid Tumors	• Biological: Allogeneic NK cells	Study Type: Interventional Phase: Phase 1	Enrollment: 18 Age: 18 Years and older (Adult, Older Adult) Sex: All	• Seoul National University Hospital • Green Cross Corporation	Study Start: September 2010 Primary Completion: August 2012 Last Update Posted: August 19, 2013	• Seoul National University Hospital, Seoul, Korea, Republic of
4	NCT00383994	Immunotherapy With NK Cell, Rituximab and Rhu-GMCSF in Non-Myeloablative Allogeneic Stem Cell Transplantation	Blood derived NK Cells	Completed No resultrs posted	• Lymphoma • Leukemia • Transplantation, Stem Cell • Lymphoid Malignancies • Disorder Related to Transplantation	• Drug: GM-CSF • Drug: Rituximab • Biological: NK Cell Infusion	Study Type: Interventional Phase: Phase 1	Enrollment: 6 Age: Child, Adult, Older Adult Sex: All	• M.D. Anderson Cancer Center • Bayer Healthcare Pharmaceuticals, Inc./Bayer Schering Pharma	Study Start: September 2006 Primary Completion: July 22, 2019 Last Update Posted: July 31, 2019	• University of Texas MD Anderson Cancer Center, Houston, Texas, United States
5	NCT00402558	Alloreactive NK Cells for Allogeneic Stem Cell Transplantation for Acute Myeloid Leukemia (AML) and Myelodysplastic Syndrome (MDS)	Completed - no results posted	Completed No results posted	• Myelodysplastic Syndrome • Leukemia	• Drug: Thymoglobulin • Drug: Busulfan • Drug: Fludarabine • Procedure: Alloreactive NK Infusion • Drug: G-CSF • Drug: Tacrolimus • Drug: Methotrexate • Drug: Interleukin-2	Study Type: Interventional Phase: Phase 1	Enrollment: 15 Age: up to 70 Years (Child, Adult, Older Adult) Sex: All	• M.D. Anderson Cancer Center	Study Start: May 2006 Primary Completion: April 2014 Last Update Posted: May 8, 2015	• UT MD Anderson Cancer Center, Houston, Texas, United States
6	NCT01853358	Phase I of Infusion of Selected Donor NK Cells After Allogeneic Stem Cell Transplantation	HLA identical allogeneic NK Cells	Completed-Phase 1-No results posted	• Hematological Malignancy	• Biological: NK Cell infusion	Study Type: Interventional Phase: Phase 1	Enrollment: 17 Age: 18 Years to 70 Years (Adult, Older Adult) Sex: All	• Institut Paoli- Calmettes	Study Start: April 2013 Primary Completion: March 15, 2018 Last Update Posted: July 12, 2018	• Institut Paoli-Calmettes, Marseille, France
7	NCT01287104	A Phase I Study of NK Cell Infusion Following Allogeneic Peripheral Blood Stem Cell Transplantation From Related or Matched Unrelated Donors in Pediatric Patients With Solid Tumors and Leukemias	Allogeneic Bone marrow NK Cells	Completed - Has results	• Leukemia • Lymphoma	• Biological: Natural Killer (NK) Cell Infusion • Biological: Stem Cell Infusion - Pag	Study Type: Interventional Phase: Phase 1	Age: 4 Years to 35 Years (Child, Adult) Sex: All	• National Cancer Institute (NCI) • National Institutes of Health Clinical Center (CC)	Study Start: January 29, 2011 Primary Completion: June 28, 2018 Last Update Posted: August 22, 2019	• National Institutes of Health Clinical Center, Five of 9 transplant recipients experienced acute graft-versus-host disease (GVHD) following aNK-DLI, with grade 4 GVHD observed in 3 subjects.
8	NCT02716571	Recruiting Blood Donor With Allogeneic Natural Killer Cell	Allogeneic natural killer cell	Completed: No results posted	• Healthy Volunteers	• Other: Leukapheresis or Plasmapheresis	Study Type: Interventional Phase: Not Applicable	Enrollment: 90 Age: 20 Years to 60 Years (Adult) Sex: All	• Seoul National University Hospital	Study Start: March 28, 2016 Primary Completion: June 2, 2017 Last Update Posted: July 31, 2017	• Seoul National University Hospital, Seoul, Korea, Republic of
9	NCT00877110	Anti-GD2 3F8 Antibody and Allogeneic Natural Killer Cells for High-Risk Neuroblastoma	Allogeneic NK Cells from a family member who shares half of the HLA proteins	Completed: No results posted	• Neuroblastoma • Bone Marrow, Sympathetic Nervous System	• Drug: cyclophosphamide, vincristine, topotecan ,allogenei NK cells & 3F8	Study Type: Interventional Phase: Phase 1	Enrollment: 71 Age: Child, Adult, Older Adult Sex: All	• Memorial Sloan Kettering Cancer Center	Study Start: April 2, 2009 Primary Completion: January 7, 2019 Last Update Posted: January 10, 2019	• Memorial Sloan Kettering Cancer Center, New York, New York, United States
10	NCT02301065	Analysis of T Cell and Natural Killer (NK) Cell in Relation to Viral Infections in Pediatric Stem Cell Transplant Patients and Donors	Blood derived FcRg-CD56+CD3- NK cells in pediatric allogeneic HSCT patients and healthy donors	Completed: No results posted	• Hematologic Malignancies		Study Type: Observational Phase:	Enrollment: 35 Age: up to 21 Years (Child, Adult) Sex: All	• St. Jude Children's Research Hospital • Michigan State University	Study Start: October 13, 2016 Primary Completion: February 6, 2017 Last Update Posted: July 17, 2017	• St. Jude Children's Research Hospital, Memphis, Tennessee, United States
11	NCT02845999	Allogenic Immunotherapy Based on Natural Killer (NK) Cell Adoptive Transfer in Metastatic Gastrointestinal Carcinoma Treated With Cetuximab	Haploidentical Natural Killer (NK) cells	Completed-No Results posted	• Gastrointestinal Metastatic Cancer	• Biological: allogenic immunotherapy based on Natural Killer cells adoptive transfer • Biological: cetuximab • Drug: Cyclophosphamide • Drug: fludarabine • Drug: interleukin-2	Study Type: Interventional Phase: Phase 1	Enrollment: 9 Age: 18 Years to 65 Years (Adult, Older Adult) Sex: All	• Centre Hospitalier Universitaire de Besancon • National Cancer Institute, France	Study Start: November 2009 Primary Completion: January 2013 Last Update Posted: July 27, 2016	• University hospital of Besançon, Besançon, France
13	NCT01181258	Penostatin, Rituximab and Ontak and Allogeneic Natural Killer (NK) Cells for Refractory Lymphoid Malignancies	Interleukin 2-activated Allogeneic Natural Killer Cells	Completed-Has results	• Non-Hodgkin Lymphoma • Chronic Lymphocytic Leukemia	• Drug: Rituximab • Biological: Interleukin-2 • Biological: Natural killer cells • Drug: Cyclophosphamide • Drug: Methylprednisolone • Drug: Fludarabine	Study Type: Interventional Phase: Phase 2	Enrollment: 16 Age: Child, Adult, Older Adult Sex: All	• Masonic Cancer Center, University of Minnesota	Study Start: August 2010 Primary Completion: September 2015Study Completion: July 2016 First Posted: August 13, 2010 Results First Posted: May 18, 2017 Last Update Posted: February 6, 2018	• Masonic Cancer Center, University of Minnesota, Observations support development of donor NK cellular therapies for advanced NHL as a strategy to overcome chemoresistance
14	NCT01105650	Allogeneic Natural Killer (NK) Cells for Ovarian, Fallopian Tube, Peritoneal and Metastatic Breast Cancer	Allogeneic donor cells	Completed-Has results	• Ovarian Cancer • Fallopian Tube Cancer • Primary Peritoneal Cancer • Breast Cancer	• Drug: Fludarabine • Drug: Cyclophosphamide • Drug: Cyclosporine • Biological: Natural killer cells • Drug: IL-2 • Drug: Methylprednisolone • Drug: Interleukin-2	Study Type: Interventional Phase: Phase 2	Enrollment: 13 Age: 18 Years and older (Adult, Older Adult) Sex: Female	• Masonic Cancer Center, University of Minnesota	Study Start: July 2010 Primary Completion: April 2014 Last Update Posted: December 28, 2017	• Masonic Cancer Center, University of Minnesota, Some adverse events reported - not published
15	NCT00586703	Safety Trial of NK Cell DLI 3-5/6 Family Member Following Nonmyeloablative ASCT	CD56-NK cells from mismatched donors	Completed-Has results	• Lymphoma	• Device: NK-CD56	Study Type: Interventional Phase: Phase 1	Enrollment: 21 Age: 18 Years and older (Adult, Older Adult) Sex: All	• David Rizzieri, MD • Duke University	Study Start: April 2005 Primary Completion: April 2013 Last Update Posted: June 12, 2014	• Duke University Health Systems" A 1-step, high-yield process is feasible, and results in high doses of NK cells infused with little toxicity. NK cell-enriched DLIs result in improved immune recovery and outcomes for some
16	NCT02118285	Intraperitoneal Natural Killer Cells and INCB024360 for Recurrent Ovarian, Fallopian Tube, and Primary Peritoneal Cancer	haploidentical donor NK cells and IL-2	Completed-No results posted	• Ovarian Cancer • Fallopian Tube Carcinoma • Primary Peritoneal Carcinoma	• Drug: Fludarabine • Drug: Cyclophosphamide • Biological: NK cells • Biological: IL-2 • Drug: INCB024360	Study Type: Interventional Phase: Phase 1	Enrollment: 2 Age: 8 Years and older (Adult, Older Adult) Sex: Female	• Masonic Cancer Center, University of Minnesota • Incyte Corporation	Study Start: July 28, 2014 Last Update Posted: December 5, 2017	• University of Minnesota Masonic Cancer Center, Minneapolis, Minnesota, United States
18	NCT00526292	Chemotherapy and a Donor Natural Killer Cell Infusion in Treating Patients With Relapsed or Persistent Leukemia or Myelodysplastic Syndrome After a Donor Stem Cell Transplant	Allogeneic NK Cells from a family member who shares half of the HLA proteins	Completed:-Has results	• Leukemia • Myelodysplastic Syndromes	• Biological: natural killer cell therapy • Drug: cyclophosphamide • Drug: fludarabine	Study Type: Interventional Phase: Phase 2	Enrollment: 12 Age: up to 120 Years (Child, Adult, Older Adult) Sex: All	• Memorial Sloan Kettering Cancer Center • National Cancer Institute (NCI)	Study Start: August 2007 Primary Completion: July 2015 Last Update Posted: February 12, 2016	• Memorial Sloan Kettering Cancer Center, New Yor: Results not conclusive as 4/6 patients showed some adverse events
19	NCT02854839	A Study of MG4101 (Allogeneic Natural Killer Cell) for Intermediate-stage of Hepatocellular Carcinoma	allogeneic Natural killer cells	Completed No results posted	• Hepatocellular Carcinoma	• Biological: MG4101	Study Type: Interventional Phase: Phase 2	Enrollment: 78 Age: 18 Years to 80 Years (Adult, Older Adult) Sex: All	• Green Cross LabCell Corporation	Study Start: November 28, 2016 Primary Completion: September 27, 2018 Last Update Posted: September 26, 2019	• Seoul National University Hospital, Seoul, Korea, Republic of • Seoul Asan Medical center, Seoul, Korea, Republic of • Samsung Medical Center, Seoul, Korea, Republic of and others
20	NCT01386619	NK DLI in Patients After Human Leukocyte Antigen (HLA)- Haploidentical Hematopoietic Stem Cell Transplantation (HSCT)	HLA haploidentical -CD3-depleted/CD56+ selected natural killer cells collected from apheresis products	Completed No results posted	• Leukemia, Myeloid, Acute • Precursor Cell Lymphoblastic Leukemia- Lymphoma • Myelodysplastic Syndromes • Lymphoma • Neuroblastoma • Rhabdomyosarcoma	• Biological: CD3- depleted/CD56+ selected natural killer cells collected from apheresis products	Study Type: Interventional Phase: • Phase 1 • Phase 2	Enrollment: 15 Age: Child, Adult, Older Adult Sex: All	• University Hospital, Basel, Switzerland	Study Start: January 2004 Primary Completion: March 2011 Last Update Posted: September 15, 2015	• Universitätsklinikum, Frankfurt, Germany • University Hospital, Basel, Switzerland
21	NCT00274846	Donor Peripheral Stem Cell Transplant in Treating Patients With Relapsed Acute Myeloid Leukemia	Peripheral Blood derived NK cells and also stem cells from the same allogeneic donor	Completed Has Results	• Leukemia	• Biological: aldesleukin • Biological: therapeutic allogeneic lymphocytes • Drug: cyclophosphamide • Drug: fludarabine phosphate • Procedure: in vitro treated peripheral blood stem cell transplantation	Study Type: Interventional Phase: Phase 2	Enrollment: 21 Age: 2 Years and older (Child, Adult, Older Adult) Sex: All	• Masonic Cancer Center, University of Minnesota	Study Start: March 2005 Primary Completion: June 2008 Last Update Posted: December 28, 2017	• Masonic Cancer Center, Minneapolis, : Supports the need to optimize the in vivo cytokine milieu where adoptively transferred NK cells compete with other lymphocytes to improve clinical efficacy in patients with refractory AML

An “off the shelf” NK cell therapy solves the one-donor, one-patient limitation that makes -autologous cell therapy processes labor-intensive. A critical step to enable allogeneic NK cell-based therapies would require a healthy donor source for NK cells and expanding to clinically relevant doses. Most clinical trials of NK cells require large numbers of cells for infusion, ranging from 5×10^6^ to 1×10^8^ CD3^neg^CD56^pos^ NK cells per kilogram body weight (5).

## Sources of Natural Killer Cells for Immunotherapy

**Figure 3:**
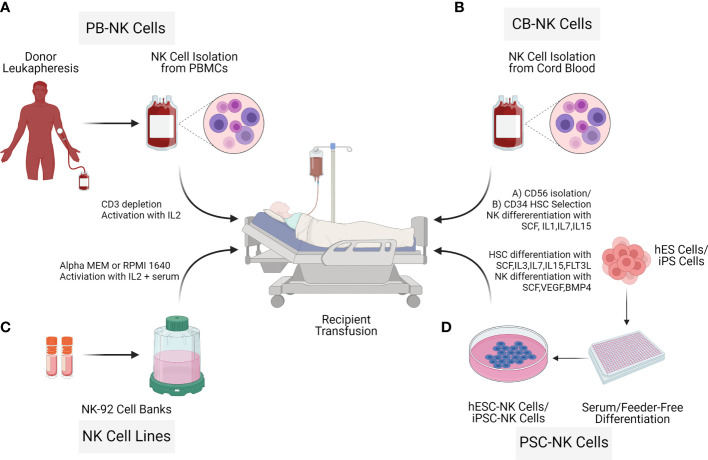
Sources of Natural killer cells for immunotherapy. NK cells for cell therapy applications can originate from different sources: Peripheral blood NK cells (PB NK cells) **(A)**, allogeneic umbilical cord blood NK cells (CB-NK Cells) **(B)**, NK cell cancer cell lines (NK-92) **(C)**, human embryonic stem cells (hESC) and inducible pluripotent stem cells (iPSCs) **(D)**. Advantages and limitations with the different NK cell sources vary as described in the NK cell isolation section.

NK cells for therapy can be acquired from various sources such as umbilical cord blood (UCB) (17), peripheral blood (PB) (18, 19), human embryonic stem cells (hESCs) or induced pluripotent stem cells (iPSCs) (20) as well as cells lines such as NK-92 (21). To date, most of the NK cell clinical trials are based on UCB-NK cells, PB-NK cells, and the lymphoma-derived NK cell line NK-92. There are critical challenges in the manufacturing process of the final therapeutic cell doses. For example, isolation and expansion of PB-NK cells and UCB-NK cells result in a mixed composition (22). The cell line NK-92 is derived from a cancer patient with non-Hodgkin lymphoma; thus, the cells need to be irradiated before infusion, limiting the NK cell persistence (23). In contrast to these limitations, hESC-NK cells and iPSC-NK cells are more homogenous and can be generated in sufficient cell numbers for allogeneic clinical use (24). Pluripotent (hESC/iPSC) derived NK cells can result in allogeneic therapy providing a standard cell-based treatment option for different diseases (24–26). Processing workflow of NK cell isolation from different donor sources through expansion for adaptive transfer is described (**Figure 3**).

### Umbilical Cord Blood

The umbilical cord is an abundant source of cytotoxic CD56^pos^CD16^pos^ NK cells, with high lytic potential of cancer cells (27). UCB-NK cells are isolated from cord blood after birth, *via* venipuncture of the umbilical cord, and purification by density gradient centrifugation (28). Alternatively, CD34 hematopoietic stem cells can be isolated from UCB and differentiated to NK cells (19, 29). NK cells generated from CD34 cells from HLA matched umbilical cord blood units showed good tolerance, no GVHD or toxicity (30). UCB is a readily available source with the potential to manufacture multiple doses from a single frozen vial of NK cells isolated from a healthy donor (21, 31). In addition, UCB NK cells are of a younger and more proliferative phenotype relative to PB NK cells (32, 33).

### Peripheral Blood

Peripheral blood contains NK cells and is a reliable source of CD34 progenitor cells from individuals undergoing GCS-F mobilization (34). Isolating large numbers of PB NK cells and hematopoietic stem cells is difficult as the percentage derived from leukapheresis can be low and highly variable (22, 35, 36). Further, cryopreservation of PB NK cells lowers the cytotoxic ability (35, 37). Allogeneic NK cells can be isolated from PBMCs by either CD3/CD19 depletion (38) or CD3 depletion and subsequent CD56 enrichment (39). The second round of purification based on CD3 depletion can also be implemented post-expansion (39) to increase NK cell purity. An evaluation of 94 samples with CD3/CD19 depletion and 13 samples with CD3 depletion/CD56 enrichments for NK cell isolations in support of 8 clinical trials demonstrated limitations and benefits with NK cell isolation strategies (34). CD3/CD19 depletion resulted in a mean NK cell recovery of 74% and viability of 96%. However, CD3 depletion/CD56 enrichment resulted in a high NK cell purity (90%), with 5% CD14 monocytes (38).

### iPSC or hESC Derived NK Cells

Pluripotent stem cells (iPSC or hESC) are an unlimited source for the derivation of human NK cells for therapy. NK cells derived from iPSC/hESC result in a homogenous population, which can be expanded on a large scale and can be genetically modified (40). NK cells are generated from different iPSC cell lines (41–43) on stromal feeders using IL-3, IL-7, IL-5, Stem cell factor (SCF), fms-like tyrosine kinase receptor-3 ligand (FLT3L) (24). NK cells derived this way are homogenous and express CD56, KIR, CD16, NKp44, NKp46, and are capable of killing tumor cells (24). Similar to iPSCs, hESCs can also be differentiated to NK cells based on stromal cell-mediated differentiation, involving CD34+CD45+ cell sorting and NK cell differentiation with IL-3, IL-5, IL-7, fms-like tyrosine kinase receptor 3 ligand (FLT3L), and Stem cell factor (SCF) (26).

Recently a stromal-free process for iPSC NK cell generation has been established based on embryoid bodies (EB) as self-stromal cells are formed inside the EB (40). Feeder-dependent hESC/iPSC was adapted to a feeder independent system before EB generation (44). To generate EBs, hESC/iPSC are seeded in APEL media containing SCF, BMP4, VEGF, Rocki (rho kinase inhibitor). In the second and final step, NK cells are generated by transferring EBs to gelatin-coated wells containing NK cell differentiation media with IL-3, SCF, IL-7, and IL-15. After four weeks of culture, differentiated NK cells stained positive for CD45 and CD56 markers were harvested (40).

### NK Cancer Cell Lines

Among the available NK cancer cell lines, only NK-92 cell line has shown antitumor activity in a variety of tumors and has worked well in pre-clinical studies (21, 45). Furthermore, NK-92 cancer cell line has received FDA approval for clinical phase patient trials (46, 47). The NK-92 cancer cell line is well characterized and robust clinical protocols are available for cGMP manufacturing (48). These cells can be genetically engineered, but with a variable efficiency of 4% - 95% (49) and expanded to substantial numbers. However, NK-92 cancer cell line requires irradiation prior to infusion, as it is cytogenetically abnormal. Select advantages and disadvantages with NK cells derived from different tissue sources are shown in **Table 2**.

**Table 2:** Advantages and drawbacks of NK Cells from different sources.

The Source of NK Cells	Advantages	Drawbacks
Peripheral Blood derived NKs (PB-NKs)	High expression of CD16+	Low number of NK Cells in PB
	Highly cytotoxic	Lower or no expression of CXCR4
	Expression of CD57, a marker of terminal differentiation of NK Cells	
NK-92 cancer cell line	Cell line product -easy to obtain	Need for irradiation before injection
	Clinically approved	Tumorigenesis potential
	CD16 negative	Safety concerns
Umbilical Cord Blood derived NKs (UCB-NKs)	High expression of CXCR4	Reduced cytotoxicity (against K562 tumor cell line)
	Minimize GvHD	Low numbers
	Ready reconstitution after transplant	Immaturity of NK cells
Placental blood derived NKs (p-NKs)	Placenta rich source for NK cells	Low cytolytic activity
	Easily, readily available	
iPSC derived NKs (iNKs)	Resource to generate unlimited numbers relevant for therapy	Complex differentiation steps
	Minimal immune rejection	Clinical effectiveness still to be proven
		Safety issues

## NK Cell Expansion for the Creation of Allogeneic Doses

Regardless of how the NK cells are sourced, every method of NK cell expansion can be classified as either a feeder-cell-based system or a feeder-free system. A multitude of cells and cell lines are used as feeders to stimulate allogeneic NK cell expansion. K562 leukemia cells have been successfully used in this regard for several decades (50) and are the most used and well-characterized example. Other examples including EBV transformed lymphoblastoid (EBV-LCL) (51), HEK293 (52), autologous irradiated PBMCs (53), Jurkat cells (54), the Wilms tumor cell line, HFWT (la5), RPMI1866 (55), MM170 (56), and Daudi (57) have also been applied with varying degrees of success. Strategies to prime and propagate NK cells using EBV-transformed lymphoblastoid cells and irradiated PBMCs continue to show promise, but protocols employing K562 cells remain superior in terms of both the magnitude and speed of expansion. Still, many groups attempt to improve the outcome even more by supplementing the culture with antibodies, such as OKT-3 (58, 59) and other cyto-stimulants, such as PHA, ionomycin (53), and concanavalin A (60). One group has even claimed an extremely robust average of 50,000-fold expansion in 21 days (about 3 weeks) using a modified K562 line that expresses membrane-bound IL-21 (61). A potential pitfall of employing feeder cells is that they are associated with a multitude of regulatory concerns. These cells must be stringently qualified using cumbersome assays and viral testing to ensure that they are free of microbial contaminants, such as mycoplasma (62). Moreover, additional actions need to be taken to ensure that the final product is free from the feeder cells. This has encouraged researchers to develop and employ several feeder-free systems in the cultivation of NK cells.

To date, there has been a clear trade-off in that feeder-free systems alleviate many regulatory concerns but result in much lower yields. Several cell-free methods can be explored to activate and stimulate NK cells, including cytokines, and antibodies. Cytokines represent the most widely studied and earliest feeder-free method for activating NK cells. IL-2 is the most potent NK cell stimulant and elicits immunostimulatory signaling, increases cytokine release (63), promotes cell motility (63), and enhances cytotoxicity (64). More recently, many alternative immunogenic cytokines have garnered attention for NK stimulation, including interleukins-15, -21, -12, -18, and -27. Much like IL-2, IL-15 stimulates NK cell proliferation, immunostimulatory receptor expression, and cytotoxicity (65, 66), which makes it a great candidate to be used as an NK stimulant in a stand-alone fashion. In addition, it boasts several benefits over IL-2. Marks-Konczalik and colleagues reported that IL-15 inhibited activation-induced cell death that results from continuous IL-2 stimulation (67)and unlike IL-2, IL-15 does not induce activation and proliferation of Tregs (68), which results in peripheral tolerance and potentially leads to a more robust anti-tumor response. However, there is a tradeoff, research conducted by Felices et al. recently demonstrated that sustained IL-15 signaling results in exhausted NK cells and a loss of *in vitro* and *in vivo* efficacy (69). Several groups have tried to stimulate NK cells with lower doses of IL-2 or IL-15 in combination with some of the other cytokines or they have developed cytokine schedules to alleviate some of the drawbacks associated with persistent stimulation with the one cytokine over the entire expansion protocol (70). IL-21 alone is not sufficient to stimulate significant NK-cell expansion (71, 72), however, there is a synergistic proliferative effect when IL-21 is combined with other immunostimulatory cytokines like IL-2 and IL-15 (71, 72). Furthermore, the addition of IL-21 to NK cell culture has been associated with increased immunostimulatory cytokine production (73) and upregulation of perforin and granzyme A and B (74), leading to enhanced NK cell cytotoxicity (75, 76). IL-12, IL-18, and IL-27 are slightly less characterized but have also displayed the ability to positively contribute to NK cell expansion, especially when used in conjunction with the IL-2 or IL-15. Research demonstrates that IL-12 can have a synergistic effect with IL-2, which results in enhanced NK cell cytokine secretion, proliferation, and cytotoxic capacity (77, 78). IL-18 and IL-27 have recently been combined with IL-15 to boost NK cell fold expansion (79). Another advantage of combining the cytokines can result in a lower dose of the individual cytokines, which can lead to a higher percentage of memory NK cells (19). The combination of IL-12, IL-15, and IL-18 drives preferential expansion of memory-like NK cells, which exhibit heightened responses when they encounter tumor cells (79–81) and longer lifespans following engraftment (79–81). An additional benefit of these cells is an increased capacity to produce immunostimulatory cytokines upon secondary challenge. This memory response is an intrinsic quality that is passed on to all cellular progeny (79–81).

Apart from these most common feeder-cell and feeder-free cytokine systems, several groups have moved towards strategies that are a hybrid of the two. Several groups have engineered feeder cells that express immunostimulatory signaling molecules, such as 41BB, IL-15 (82, 83), and IL-21 (63, 84–86) on their cell surfaces. These strategies have resulted in highly cytotoxic NK cells that display both extremely high proliferative capacities (up 50,000-fold expansion) (61), extended survival, and the ability to secrete immunogenic cytokines, leading many groups to adopt these methods into their clinical protocols. This approach has recently been taken one step further to avoid safety concerns by stimulating NK cells with K562-mb21-41BBL cell lysates (87).

**Figure 4:**
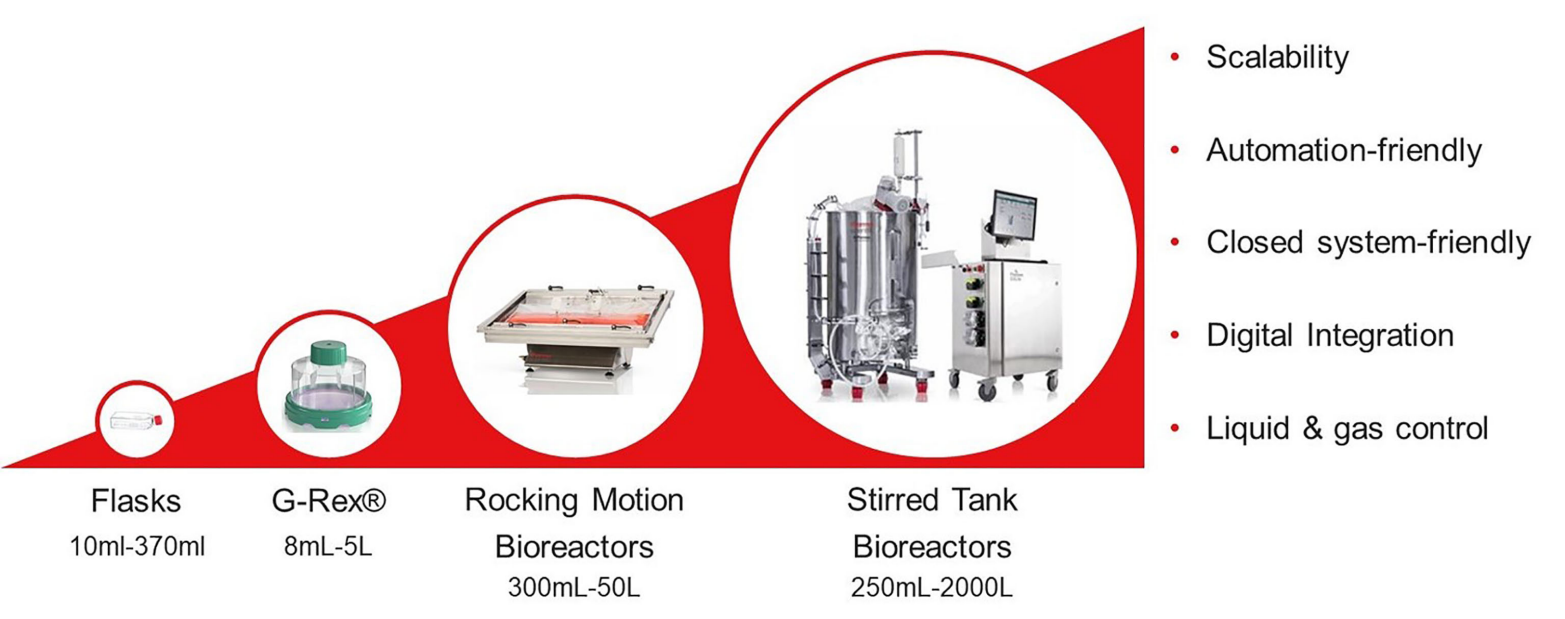
Bioreactors offer several advantages to the clinical manufacturing of cell therapies. The shift from static vessels on the left toward dynamic bioreactors on the right allows for several process improvements, such as scalability, automated and closed operation, digital integration, and intimate control of liquids. These capabilities result in increased safety and consistency, reduced labor requirement and cost, and improved quality of cellular output.

Most of the experiments and trials discussed in this review have utilized small-scale, open methods for NK cell activation and expansion, such as flasks and G-Rex vessels. However, these methods are hampered by logistical hurdles, inconsistencies, and safety concerns. To reach the desired cell numbers for allogeneic manufacturing and clear all regulatory and safety hurdles associated with drug approval, it will be necessary to develop closed, and automated systems with large-scale capabilities. Hence, clinical scale NK cell manufacturing development suitable for effective allogeneic therapy production is a priority. Several options have been explored, including a G-Rex-based method that was developed under good manufacturing practice (GMP) conditions and required little to no manual intervention for the 8- to 10-day expansion and yielded 19 billion functional NK cells (88). Another example of static culture is the use of large, gas-permeable culture bags, which were successfully applied in combination with feeder cells, antibodies, and cytokines to yield an NK cell fold expansion of 15,000 (89). A more recent trend for achieving clinical scale NK cell expansion has been the use of bioreactors. In addition to large cell capacity, these devices are highly adaptable for closed and automated manufacturing processes (**Figure 4**). Robust NK cell expansion with the Xuri Cell Expansion System W25 (Cytiva) has been demonstrated by several groups (90–92). The most common approach is to expand the isolated NK cells in static culture before transferring them to rocking bioreactors, which effectively nourish high cell densities (90–92). These workflows were able to generate 50 billion highly cytotoxic NK cells (91). Stirred tanks are another type of dynamic culture bioreactor that has gained favor in the NK cell therapy community. Pierson and colleagues first demonstrated that the cultivation of NK cells in a 750ml-stirred tank significantly outperforms that in a comparable static vessel (93). Moreover, it was recently shown that NK cell propagation in 2L stirred tanks scaled up exceptionally well to 50L stir tanks (94) making this platform an excellent fit for allogeneic manufacturing workflows. Aside from the well-known wave motion reactors and stirred tank reactors, there has also been success using lesser-characterized reactors, such as the ZRP Bioreactor 50M, which was able to grow massive amounts of highly pure and functional NK cells (95).

## NK Cell Therapy Packaging and Release Testing

Once the desired expansion is achieved, a major challenge is the downstream processing of these cells and preparing the allogeneic doses. Manufacturing and storing these “off-the-shelf” doses remotely, requires cryopreservation, which is often problematic in the case of NK cells. In addition to a loss in cell viability, it is common to see a significant drop in cytotoxicity after thawing. This functional loss routinely corresponds to a reduction in the expression of CD16 on NK cell surfaces (63). However, many groups are attempting to mitigate these issues with different strategies. A few more promising examples are to expose thawed cells to IL-2 immediately, thereby restoring their cytotoxic capacity (60), using twice as many cells in the dose to compensate for the reduced function per cell (96), and inoculating the NK cells immediately after thawing them (37, 96). A separate, but related concern, is a 6-fold decrease in motile NK cells following cryopreservation (37). Efforts to develop effective cryopreservation solutions that preserve NK cell numbers and functionality are currently a priority to carry this field forward.

**Figure 5:**
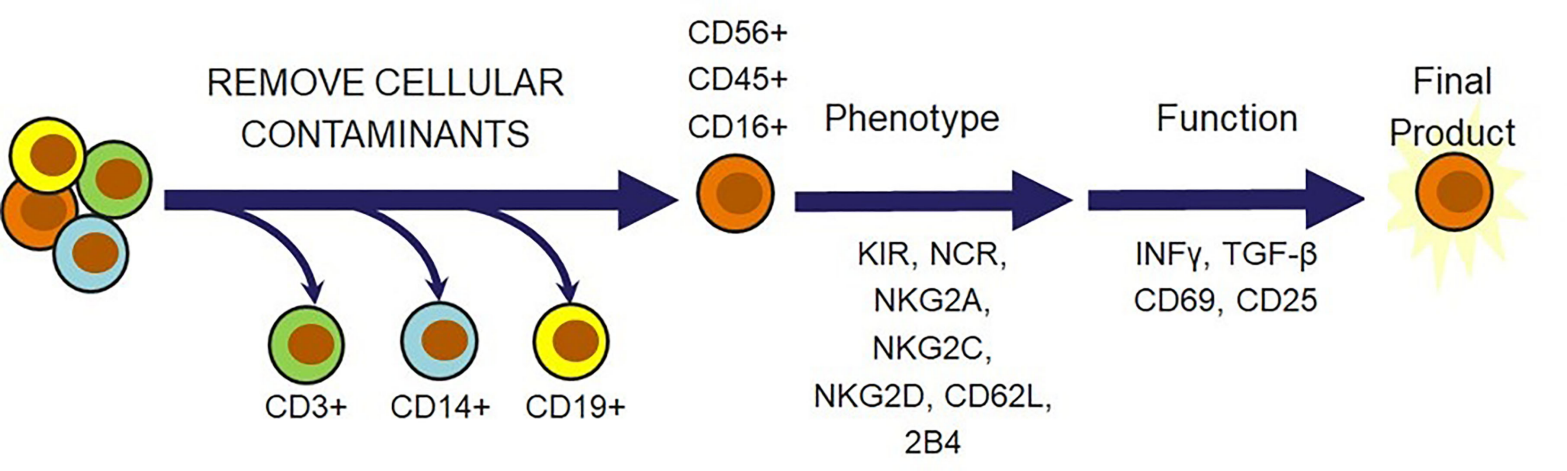
Natural killer cell-specific strategies for NK cell therapy release criteria. In addition to verifying that cell therapies are free from endotoxins, mycoplasma, bacteria, and feeder cells, there is a multitude of cell markers that can be selected to ensure that the therapeutic population possesses desired phenotypic and functional qualities. T cells, monocytes, and B cells must be removed for safety. Receptors and cytokines can then be evaluated to confirm that the outgoing cell population is responsive, cytotoxic, and safe.

Beyond viability and cytotoxicity issues following the cryopreservation and recovery cycle, there is a multitude of other criteria that should be considered before confidently releasing the NK cells for administration as a therapeutic dose. Safety is the overarching theme for most of these considerations. Several of these requirements are focused on confirming that there are no undesirable trespassers in the dose, such as endotoxins, mycoplasma, bacteria, or feeder cells if they were used for expansion. Confirmation that the dose consists of the desired cellular population is also highly important in preventing the onset of adverse effects that these cellular contaminants can cause. This can be done by setting a minimum requirement for the percent of CD56^pos^/CD3^neg^ cells and a maximum allowed amount of CD3^pos^ T cells, CD19^pos^ B cells, and CD14^pos^ monocytes that can safely be released in a dose. These are the key regulatory principles that agencies across different geographical locations will require for cell therapies. Several additional ideas could be incorporated to further ensure therapeutic efficacy. An example of this could be flow cytometric characterization of activating receptors, such as NCRs, NKG2D, NKG2C, NKG2E, 2B4 and the inhibitory receptors NKG2A and KIRs. In addition, indicators of cytotoxic capabilities, CD16 and CD25, markers of differentiation status, CD62L, CD45, HLA-DR, CD69, and CD57, and functional analysis of IFNγ or TGF-β can be included (20) (**Figure 5**). It may also be beneficial to modify the cellular requirement based on the characteristics of the disease state. The tumors and surrounding microenvironments pose significant obstacles that are directly opposed to the proper function of adoptive cell therapies, such as NK cell therapies (97). While many of the escape mechanisms are identified, there is often no way to identify which ones a particular tumor is employing. Thus, understanding the individual challenges associated with each tumor through a standardized molecular imprint could go a long way in cultivating the most effective cell therapy or combination therapy.

## CAR-NK Engineering

When NK cells are engineered with a tumor-specific chimeric antigen receptor (CAR), superior NK cell elicited cytotoxicity and improved cell infiltration into the tumor microenvironment are noticed. Genetic modification of NK cells by transducing with CAR receptors directed against tumor specific antigens may enhance both NK cell tumor specificity and NK cell persistence. CARs are engineered receptor proteins that recognize a target antigen on tumor cells and are successfully used in T cell therapy for lymphoid leukemias. Most of the CAR-T trials are restricted to autologous therapies, which are cumbersome, although strikingly efficient in targeted tumor cell killing (98). The development of allogeneic CAR-T cells is challenging, as these treatments must be specifically tailored to avoid graft *versus* host diseases (GVHD) and elimination by the host immune system (99). In contrast, several advantages are recognized with CAR-NK cell therapy over CAR-T cell therapy clinical approaches. First, there are less side effects such as low/no GVHD (100), cytokine release syndrome (101) and neurotoxicity (102). Second, CAR-NK cells can also eliminate tumor cells efficiently in a CAR-independent manner through their stimulatory and inhibitory receptors and CD16-mediated ADCC (103). Therefore, several researchers are exploring different approaches to genetically engineer NK cells with CARs to augment the efficiency of NK cells to kill tumors (104).

**Figure 6:**
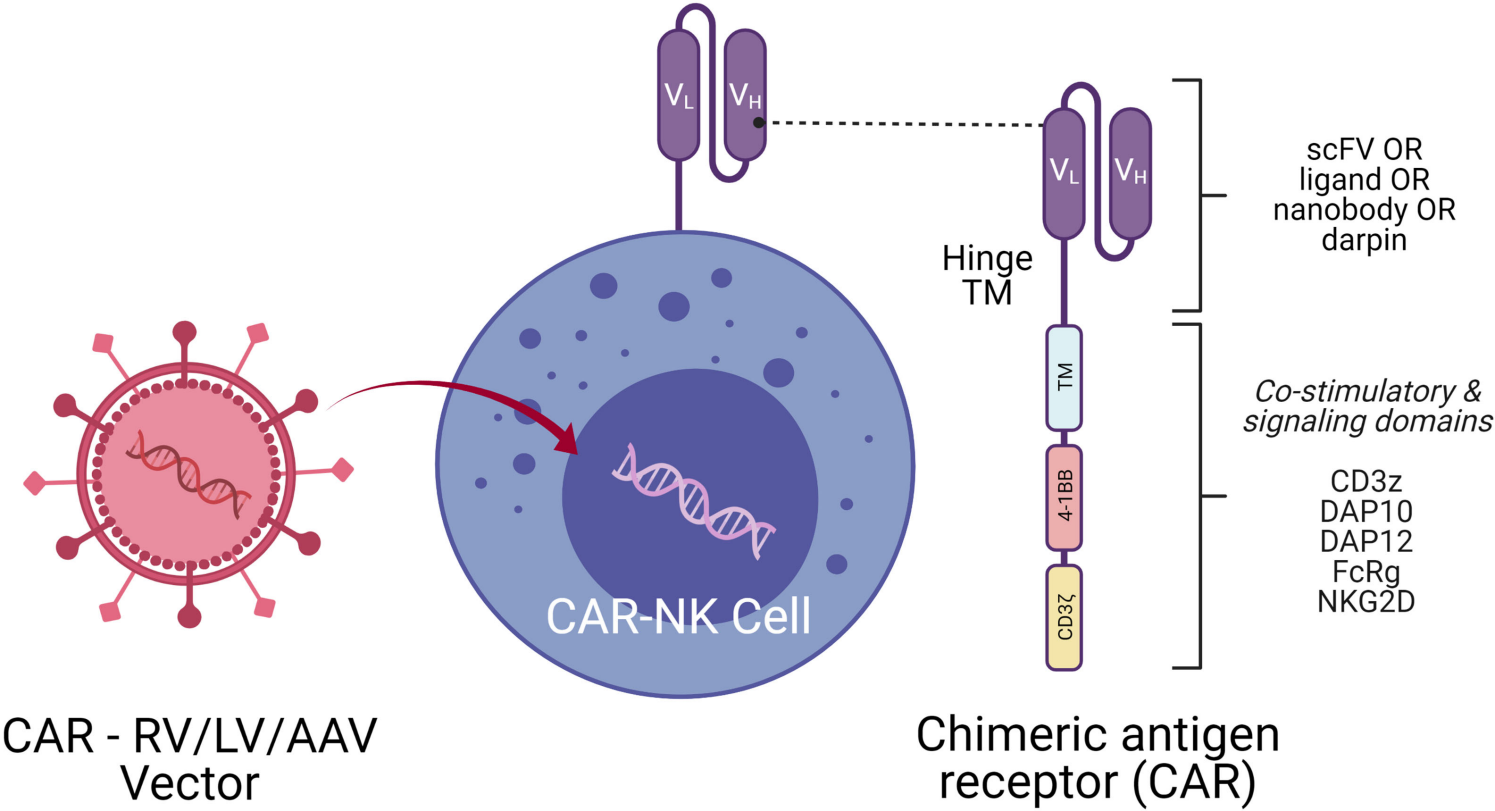
CAR-NK Molecule Delivery of genetic cargo into NK cells with CAR encoding retro (RV), lenti (LV), or adeno-associated (AAV) vectors. CAR molecule is shown on the right side with single-chain variable fragment (scFv including V_H_ and V_L_ chains), hinge, transmembrane (TM), and signaling domain. Co-stimulatory signaling domains are indicated in different colors.

NK cells are successfully engineered to express CARs against several tumor-specific antigen targets and are shown to be efficient for *in vitro* and *in vivo* killing of tumor cells in experimental investigational studies. Human iPSC-derived NK cells engineered with specific CAR constructs demonstrated significantly enhanced targeted anti-tumor activity in an ovarian cancer xenograft model (105). Although autologous NK cells can be generated *in vitro*, they have limited efficiency against own patient’s tumor cells. There are currently 72 clinical trials using CAR-NK cell lines and 35 primary CAR-NK preclinical studies based on PubMed and Global data (www.carnkreview.com) targeting different tumors (106). However, only 5 studies are ongoing in phase I & II clinical trials at www.clincaltrials.gov (**Table 3**). CAR constructs for NK cells consist of three domains: an extracellular antigen recognition domain, a transmembrane domain, and an intracellular cytoplasmic signaling domain (**Figure 6**). The ectodomain contains a single-chain variable fragment (scFv) derived from an antibody recognizing the tumor antigen. The transmembrane domain anchors the CAR structure to the effector cell membrane. CAR recognition of specific antigen triggers intracellular activation domain that results in the killing of the target cells. From the limited number of CAR-NK trials so far, no significant adverse events are noted, and the CAR-NKs showed robust cytolytic activity. In the CAR-NK trials that fit the allogeneic and off-the-shelf approach, CAR-NK cells from a single healthy donor were expanded in cell culture for appropriate dosing. The infused CAR-NKs persisted and expanded at a low level, based on PCR results, for a year within the tumor microenvironment in an ablative conditioning regimen. Most engineered CAR-NK cells are directed against blood-related malignancies, such as CD19 for B cell lymphomas, CD22 for refractory B-cell lymphomas and solid tumors, NKG2D-ligand for pancreatic cancers, and CD33 and ROBO1 specific BiCAR-NK/T for malignant and metastatic solid tumors (107). Barriers to a successful implementation of CAR-NK in solid tumors are recently reviewed, including off-tumor effects, impaired antigen recognition, poor cell trafficking, harsh tumor environment, and immune evasion (108).

**Table 3:** On-going CAR-NK Clinical Trials.

	NCT Number	Title	Status	Conditions	Source of NK Cells	Interventions	Clinical trial phase	Population	Sponsor/ Collaborators	Locations
1	NCT04324996	A Phase I/II Study of Universal Off-the-shelf NKG2D-ACE2 CAR-NK Cells for Therapy of COVID-19	Recruiting	• COVID-19	Cord blood :NKG2D CAR- NK cells,ACE2 CAR-NK cells,NKG2D-ACE2 CAR-NK cells	• Biological: NK cells,IL15-NK cells,NKG2D CAR- NK cells,ACE2 CAR-NK cells,NKG2D-ACE2 CAR-NK cells	Study Type: Interventional Phase: • Phase 1 • Phase 2	Enrollment: 90 Age: 18 Years and older (Adult, Older Adult) Sex: ll	• Chongqing Public Health Medical Center • Chongqing Sidemu Biotech • Zhejiang Qixin Biotech	• Chongqing Public Health Medical Center, Chongqing, China
2	NCT03940833	Clinical Research of Adoptive BCMA CAR-NK Cells on Relapse/Refractory MM Study Documents:	Recruiting	• Multiple Myeloma	Engineered NK-92 Cells	• Biological: BCMA CAR-NK 92 cells	Study Type: Interventional Phase: • Phase 1 • Phase 2	Enrollment: 20 Age: 18 Years to 80 Years (Adult, Older Adult) Sex: All	• Asclepius Technology Company Group (Suzhou) Co., Ltd.	• Department of Hematology, Wuxi People's Hospital, Nanjing Medical University, Wuxi, Jiangsu, China
3	NCT03940820	Clinical Research of ROBO1 Specific CAR-NK Cells on Patients With Solid Tumors Study Documents:	Recruiting	• Solid Tumor	ROBO1 Specific CAR-NK Cells	• Biological: ROBO1 CAR-NK cells	Study Type: Interventional Phase: • Phase 1 • Phase 2	Enrollment: 20 Age: 18 Years to 75 Years (Adult, Older Adult) Sex: All	• Asclepius Technology Company Group (Suzhou) Co., Ltd.	• Radiation Therapy Department, Suzhou Cancer Center, Suzhou Hospital Affiliated to Nanjing Medical University, Suzhou, Jiangsu, China
4	NCT04887012	Clinical Study of HLA Haploidentical CAR-NK Cells Targeting CD19 in the Treatment of Refractory/ Relapsed B-cell NHL Study Documents:	Recruiting	• B-cell Non Hodgkin Lymphoma	HLA haploidentical CAR-NK cells targeting CD19	• Biological: anti- CD19 CAR-NK	Study Type: Interventional Phase: Phase 1	Enrollment: 25 Age: 18 Years to 75 Years (Adult, Older Adult) Sex: All	• Second Affiliated Hospital, School of Medicine, Zhejiang University	• 2nd Affiliated Hospital, School of Medicine, Zhejiang University, Hangzhou, Zhejiang, China
5	NCT05020678	NKX019, Intravenous Allogeneic Chimeric Antigen Receptor Natural Killer Cells (CAR NK), in Adults With B-cell Cancers Study Documents:	Recruiting	• Lymphoma, Non- Hodgkin • B-cell Acute Lymphoblastic Leukemia • Large B-cell Lymphoma • Mantle Cell Lymphoma	allogeneic CAR NK cells targeting CD19	• Biological: NKX019	Study Type: Interventional Phase: Phase 1	Enrollment: 60 Age: 18 Years and older (Adult, Older Adult) Sex: All	• Nkarta Inc.	• Colorado Blood Cancer Institute, Denver, Colorado, United States • Peter MacCallum Cancer Center, Melbourne, Victoria, Australia

## Delivery Systems to Engineer NK Cells

**Figure 7:**
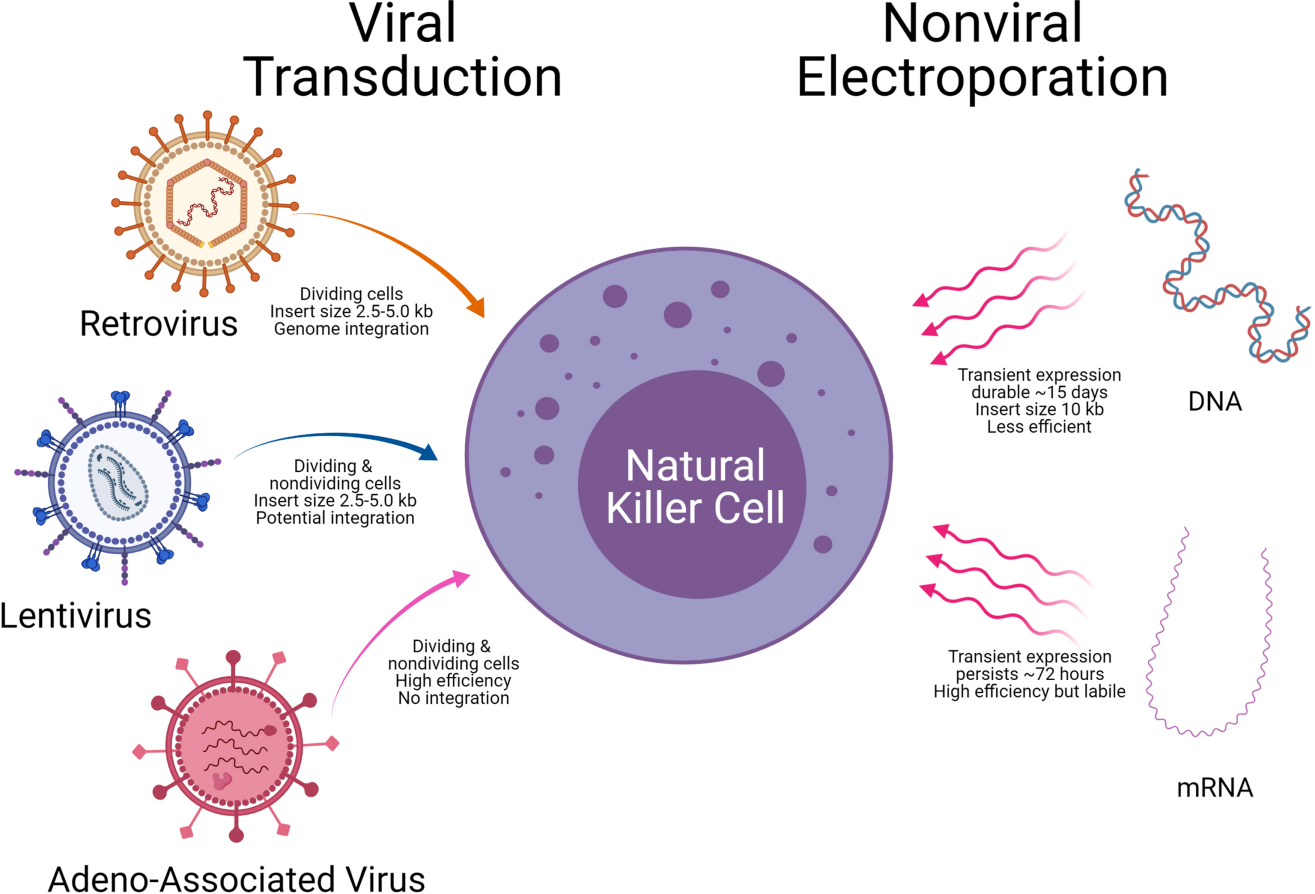
Delivery technologies to engineer Natural killer cells. Modes of genetic cargo delivery into NK Cells by viral transduction and non-viral electroporation for gene engineering of NK Cells. Specific advantages and limitations are noted below the arrows.

A critical aspect of CAR-NK generation is the introduction of genetic elements into NK cells, referred to as CAR-NK engineering. Once the genetic element is introduced into NK cells, the subsequent expansion of the CAR-NK cells with the cytotoxic killing of the target tumor cell will be another important consideration. The introduction of genetic material into NK cells is carried out using either viral vectors (retrovirus, lentivirus, and Adeno associated virus) or non-viral methods (mRNA and DNA). Examples of the viral and non-viral vector delivery systems with select pros and cons are shown in **Table 4** and **Figure 7**.

**Table 4:** Advantages and disadvantages with different gene delivery vectors.

Vector	Advantages	Drawbacks
**Viral Vectors**		
Adenovirus	Deliver large dsDNA (~8kb)	Transient expression Elicit immune response
Adeno-associated virus	Deliver to dividing and nondividing cells ssDNA (~4kb)	Difficulty producing vectors Limited transgene Elicit immune response
Retrovirus	Deliver to dividing cells Sustained vector expression ssRNA (~8kb)	cannot transfect non-dividing cells Low transfection rate *in vivo* Elicit immune response Risk of insertion
Lentivirus	Deliver to non-dividing cells Genome integration into host ssRNA (~8kb)	Possibility for insertional mutagenesis
**Non-Viral Vectors**		
	Less/No insertional mutagenesis Low/No immunogenicity Can scale-up Can be chemically modified Relatively less expensive	Less effective Transient expression

### Retroviral Vector Systems

Recent studies from the Rezvani laboratory at MDACC used retroviral vectors to deliver anti-CD19 CAR into NK cells along with IL-15 and inducible caspase 9. The CAR NK cells were used to treat CD19 positive tumors; 7 of 11 patients (64%) had a complete response (4 of 5 patients with CLL and 4 of 6 with non-Hodgkin’s lymphoma). All 8 patients had an objective response (73%) at 13.8 month follow-up (109). Gene transfer did not change the function or phenotype of NK cells, nor did it change the proliferative or cytotoxic ability post engineering. Another recent study used retroviral vector systems that ectopically expressed iC9/CAR.19/IL15 to generate CAR-CD19-NK cells from cord blood that persisted for a long time in the tumor microenvironment (110, 111). In both studies, the retroviral vector ectopically produced IL15 that is crucial for NK cell survival and conditionally expressed a caspase 9 (iC9)- an inducible suicide gene that could be activated to shut off the system by eliminating transduced cells when needed. Though retroviral vector systems have high transfection efficiency, the cDNA can integrate into the NK cell genome causing insertional mutagenesis and sometimes an induction of immune response (112).

### Lentiviral Vector Systems

Lentiviral transduction is the preferred choice to modify NK cells. The lentiviral method allows transduction of primary and non-activated NK cells, and unlike the retroviral vector system, does not require dividing cells (113). Single lentiviral transduction usually results in lower transduction efficiencies, PB NK (<10%) or CB NK (<30%) (114). In Japan, a study led by Dr. Ueda used a lentiviral system to express CAR-NK-GPC3 for solid tumors of hepatocellular carcinoma (HCC) and ovarian cancers that are treated in *in vivo* animal models with good success (115). Recent studies have improved the efficiencies of lentiviral delivery, by using statins to upregulate the low-density lipoprotein (LDL) receptor on NK cells enhancing the transduction efficiency by 30-50% (116).

Pseudotyped lentiviral particles are glycoproteins derived from other enveloped viruses that enable the tropism of the lentiviral. The ability to generate CAR-NK cells depends on the envelope protein lentivirals express. Vesicular stomatitis virus G glycoprotein (VSV-G) pseudotype particles showed the highest transduction efficiency of primary NK cells compared to retroviral vectors (117). Feline endogenous retrovirus envelope protein RD114-TR was similar to VSV-G pseudotype particles for primary human NK cells (118). Further, a Baboon envelope pseudotyped lentiviral vector BaEV-LV was significantly better than both the RD-114-TR and VSV-G pseudotyped lentiviral vector (119). Choosing which LV pseudotypes VSV-G *versus* RD114-TR *versus* BaEV-LV has the best transduction efficiency should be an essential consideration for CAR-NK generation. The advantages and disadvantages of different LV pseudotypes have been recently reviewed (120, 121).

### Adeno Associated Viral Delivery

Alternative viral vectors with a better safety profile are adenovirus-associated virus (AAV) vectors. One way to improve the efficiency of NK cell cytotoxicity is by blocking their inhibitory receptors. Using CRISPR/cas9 driven delivery by recombinant adeno-associated virus serotype6 (rAAV6), highly efficient knockout of A Disintegrin and Metalloproteinase-17 (ADAM17) and programmed cell death 1 (PDCD1) genes in NK cells was accomplished (121). KO of ADAM17 and PDCD1 improved NK activity, cytokine production and cancer cell cytotoxicity. These approaches demonstrate an easy-to-use strategy for efficient gene editing and delivery with AAV vector systems for NK cell therapies (121). However, one limitation with AAV is its packaging capacity (~5kb) that limits a large gene transfer. NK cells in general have a low propensity for viral transduction, and higher cell death. Hence, commercially available viral transduction enhancers such as LentiBOOST, PGE2, PS, Vectofusin-1, ViraDuctin, Retronectin, Stauro and 7-hydroxy stauro are sometimes employed to improve vector transduction.

### Non-Viral DNA Transfection and mRNA-Electroporation

Successful electroporation of DNA into the NK-92 cancer cell line was shown, but not in primary NK cells from PBMCs or cord blood (33). Recently, an improved method with NK cells expanded with IL-2 was reported with 40% efficiency of DNA plasmid transfer (122). Following DNA transfer by electroporation, the viability of NK cells was lower, due to harsh electroporation conditions, and the DNA transfection efficiency was less compared to resting NK cells. The real advantage of this approach is complex constructs can also be transferred efficiently into the cells. Plasmid DNA of small (~3.5Kb) and large sizes (~12.5Kb) are transferred with a substantial increase of transfection up to 5-fold compared to the standard electroporation approach (123).

Some researchers are exploring electroporation to express CAR molecules on NK cells (124, 125). Unlike DNA electroporation, mRNA electroporation of NK cells may be an efficient alternative, but it induces only transient expression of the transferred gene. mRNA electroporation efficiencies are usually high (80-90%) for PBMCs or cord blood cells and require cytokine stimulation such as IL-2 for post-transduction expansion or the use of feeder cells that are engineered to secrete IL2 for better viability of cells (126). Transfection efficiency with mRNA by electroporation depends on the dose of mRNA (25-200 ug/ml) (127). High dose of mRNA results in poor viability of cells following transfection. In general, post electroporation expansion is contraindicated with mRNA approaches as it leads to dilution of the mRNA.

Recently another charge-based chemical method has been tried successfully to deliver CAR mRNA into non-dividing NK cells using a nucleofection approach that showed high efficiency (128). A specific advantage of using mRNA delivery system is the transient expression of protein by mRNA, thus avoids the risk of genome integration, least expensive to manufacture and savings of time (129). Another strategy that has been less frequently used for stable non-viral gene delivery is employing DNA transposons to transduce NK cells which is cost effective, has large cargo (ex: CAR in combination with activating receptors or cytokines) deliver capacity with stable integration. Their disadvantages include potential insertional mutagenesis and the transposon must be delivered as DNA (130, 131). Despite the limitations described above, the most successful non-viral gene delivery for primary NK cells is still rapid transient expression by electroporation.

### Engineering NK Cell Receptors

For CAR-engineered cells to act, identifying specific tumor antigens as targets is a challenge, whether for T cells or NK cells. Human NK cells have innate inhibitory receptors such as KIRs and NKG2A molecules that recognize MHC class I and evoke response through immunoreceptors tyrosine-based inhibitory motif (ITIM). T cell immunoreceptor with immunoglobulin and ITIM domain (TIGIT) is an inhibitory receptor expressed on T and NK cells and is a promising emerging target for cancer immunotherapy. TIGIT interacts with ligands CD115 and CD112 expressed on tumor cells. There is evidence that TIGIT blockade can help tumor regression (132).

In contrast, activating receptors on NK cells such as NKG2D and DNAX accessory molecule 1 (DNAM-1) play a crucial role in tumor surveillance since this receptor has a wide range of ligands that provide target specificity (133). Preclinical study data using CAR-NKG2D is promising in colorectal cancer patients (124) and multiple myeloma patients (134). Natural cytotoxicity receptors (NCRs) like NKp30, NKp44, and NKp46, can recognize multiple stress ligands in infection and oncogenic transformation. Harnessing these receptors on NK cells and their ligands on tumor cells is another new strategy to create CAR-NK cells that can induce anti-tumor immunity.

With the advances made in viral and non-viral gene delivery approaches to generate better CAR-NK molecules, there will be a heightened focus on how these cells perform in clinical trials over the next few years. These results will help determine whether CAR-NKs can effectively target and kill tumor cells (135). The viability of CAR-NK cells in the tumor microenvironment is central to the success of therapy, in addition to the repeated dosing of the cells. CAR engineering of NK cells primarily resides between two choices of stable high expression by viral vectors or rapid transient expression of non-viral delivery systems using electroporation.

## Conclusions and Perspectives

NK cells are critical in immune surveillance of invading viruses and kill tumor cells without the need of tumor specific antigen presentation. Pre-clinical data from early phase clinical trials has significantly increased our knowledge for the use of allogeneic donor NK cells across a wide range of hematological malignancies and solid tumors. Recent advances include developing NK cell expansion protocols without the use of feeders, serum, activation technologies, validation of NK cells from different tissue sources, ability to selectively use donor NK cells with minimal HLA matching, genetic modification to create CAR-NK constructs, and transfer of genetic material using viral and nonviral delivery technologies. These advances point towards a true “off-the-shelf” NK cell therapy. Despite impressive advances, there are multiple outstanding challenges with NK cell therapies. Methods following good manufacturing practices to generate large clinical doses from a single healthy donor and selective expansion appropriate NK cell subsets with best predictive KIR/HLA are needed. Additionally, tumor immune evasion remains a large barrier. Once the NK or CAR-NK cells are infused into the patient, the long-term persistence of these cells *in-vivo* in the tumor microenvironment needs to be ensured and monitored. Another limitation with NK and CAR-NK cells is the memory property *in vivo*, which is not fully understood as in the case of memory T cells in adaptive immunity. Identifying novel CAR targets and generation of NK specific CAR constructs will enable CAR-NK cell homing and persistence in solid tumors contributing to breakthrough approaches driving allogeneic NK therapies towards the next frontier of cancer immunotherapy.

## Author Contributions

EH, EZ, TS, AH, NK and MCV wrote the manuscript and, SB prepared figures. All authors contributed to the article and approved the submitted version.

## Conflict of Interest

EH, EZ, TS-N, AH, SB, NK, and MV had compensated employment at Thermo Fisher Scientific.

## Publisher’s Note

All claims expressed in this article are solely those of the authors and do not necessarily represent those of their affiliated organizations, or those of the publisher, the editors and the reviewers. Any product that may be evaluated in this article, or claim that may be made by its manufacturer, is not guaranteed or endorsed by the publisher.
